# Intrinsic functional connectivity among memory networks does not predict individual differences in narrative recall

**DOI:** 10.1162/imag_a_00169

**Published:** 2024-05-20

**Authors:** Kyle Kurkela, Maureen Ritchey

**Affiliations:** Department of Psychology and Neuroscience, Boston College, Chestnut Hill, MA, United States

**Keywords:** episodic memory, default network, functional connectivity, individual differences

## Abstract

Individuals differ greatly in their ability to remember the details of past events, yet little is known about the brain processes that explain such individual differences in a healthy young population. Previous research suggests that episodic memory relies on functional communication among ventral regions of the default mode network (“DMN-C”) that are strongly interconnected with the medial temporal lobes. In this study, we investigated whether the intrinsic functional connectivity of the DMN-C subnetwork is related to individual differences in memory ability, examining this relationship across 243 individuals (ages 18-50 years) from the openly available Cambridge Center for Aging and Neuroscience (Cam-CAN) dataset. We first estimated each participant’s whole-brain intrinsic functional brain connectivity by combining data from resting-state, movie-watching, and sensorimotor task scans to increase statistical power. We then examined whether intrinsic functional connectivity predicted performance on a narrative recall task. We found no evidence that functional connectivity of the DMN-C, with itself, with other related DMN subnetworks, or with the rest of the brain, was related to narrative recall. Exploratory connectome-based predictive modeling (CBPM) analyses of the entire connectome revealed a whole-brain multivariate pattern that predicted performance, although these changes were largely outside of known memory networks. These results add to emerging evidence suggesting that individual differences in memory cannot be easily explained by brain differences in areas typically associated with episodic memory function.

## 1 Introduction

Have you ever spoken with a family member about an event that happened long ago and admired the level of detail with which they could recall the event? While important differences exist at the extreme ends of the spectrum of memory ability, such as in patients with hippocampal damage (e.g., Patient H.M.;Corkin, 2002) or in individuals with superior autobiographical memory (LePort et al., 2012;Parker et al., 2006), experiences like these reinforce that there is substantial variation within the general population in the ability to recall the details of past events. Yet relatively little is known about the brain processes that explain such individual differences among healthy adults. In this study, we investigated the relationship between individual differences in memory performance and functional communication among regions known to be involved in episodic memory.

Prior studies examining the relationship between functional connectivity and individual differences in memory have largely focused on the functional connectivity of the hippocampus due to its important role in memory function (Bird & Burgess, 2008;Corkin, 2002;Squire, 1992). These studies have generally found that increased hippocampal functional connectivity is associated with better memory ability, including its bilateral functional connectivity (L. Wang, Negreira, et al., 2010) and its functional connectivity with cortical areas such as the lateral occipital cortex (Tambini et al., 2010) and the posterior medial cortex (Touroutoglou et al., 2015;L. Wang, LaViolette, et al., 2010). This positive relationship between hippocampal connectivity and memory ability can be interpreted as reflecting greater responsiveness of the hippocampus to time-varying signals across the brain in individuals with better memory, or conversely, disruption in hippocampal communication in individuals with worse memory. However, these studies have often been underpowered, both in terms of the number of participants collected (Marek et al., 2022) and in terms of the amount of data collected per participant (Anderson et al., 2011;Gordon, Laumann, Gilmore, et al., 2017;Laumann et al., 2015). Brain-wide association studies, for example, require thousands of participants to achieve acceptable levels of statistical power (Marek et al., 2022), though this problem is mitigated by using a region of interest-based approach to reduce the number of comparisons or by focusing on multivariate patterns within the connectome. Functional connectivity studies may additionally benefit from more data being collected per participant, as it has been recently shown that at least 30 minutes of high-quality MRI data is required to achieve good levels of reliability of the functional connectome (i.e.,*r*> 0.85;Gordon, Laumann, Gilmore, et al., 2017). This is important because the reliability of a measurement places a key constraint on the measurable effect size of the correlation between two constructs of interest (e.g., functional connectivity and behavior). Most existing studies relating functional connectivity to memory ability have typically used data from one MRI scan, often comprising 6-8 minutes of data.

Because prior research has largely focused on the functional connectivity of the hippocampus, relatively less is known about how functional connectivity among cortical networks relates to episodic memory. Episodic memory has been linked to the functions of the default mode network (DMN), a set of brain regions that tend to be co-activated during rest and during tasks involving episodic construction (Buckner & DiNicola, 2019;Buckner et al., 2008;Ritchey & Cooper, 2020). In particular, task-based MRI studies have shown that memory tasks recruit a ventral subnetwork of the DMN that is strongly interconnected with the medial temporal lobes (Andrews-Hanna et al., 2010;Buckner & DiNicola, 2019;Barnett et al., 2021). This DMN subnetwork, labeled DMN-C in recent brain parcellations (Schaefer et al., 2018;Yeo et al., 2011), consists of the retrosplenial cortex, parahippocampal cortex, and the posterior angular gyrus. The DMN-C subnetwork is commonly co-activated with an adjacent, more dorsal DMN subnetwork, labeled DMN-A, which consists of medial frontal and parietal regions. Recent work has shown that these two subnetworks are dissociable in terms of their functional connectivity during event perception (Cooper et al., 2021) as well as their contributions to memory retrieval (Kurkela et al., 2022). In the latter study, when activity estimates from both ventral and dorsal DMN regions were included in a model predicting retrieval success, only the ventral regions significantly predicted success in retrieving event-specific associations. Based on these findings, we hypothesize that the function of the DMN-C subnetwork may be central in determining individual differences in episodic memory ability, and in particular, the ability to recall specific details associated with past events.

More recent experiments have looked at the relationship between memory and intrinsic functional connectivity using larger samples (Przeździk et al., 2019) or incorporating a broader set of brain regions (Sneve et al., 2017;van Buuren et al., 2019). These studies suggest that there is a complex, distributed pattern of whole-brain functional connectivity beyond the hippocampus that is related to individual differences in episodic memory ability, but the exact nature of this relationship has varied from study to study. Some studies suggest that superior rememberers have default mode networks that are decoupled from perceptual regions of the brain (Sneve et al., 2017), whereas others suggest that superior rememberers are characterized by decreased connectivity within the ventral default mode network and strong connectivity between the dorsal default mode network and the frontal-parietal control network (van Buuren et al., 2019). Other work suggests that memory ability is associated with hippocampal-cortical connectivity patterns that gradually change along the hippocampal long axis (Przeździk et al., 2019). Taken together, these studies suggest that there is likely some whole-brain functional connectivity pattern that is related to episodic memory ability, but the exact nature of this whole-brain pattern remains unclear.

In the present study, we investigated the relationship between intrinsic functional connectivity and episodic memory ability, focusing on narrative recall as a hallmark of intact episodic memory that has been linked specifically to the function of the DMN (Lee et al., 2020;Ritchey & Cooper, 2020). This study improved on previous work by incorporating a relatively large sample size and by incorporating measures of “generalized functional connectivity” that increase the amount of functional data included per participant, which improves the reliability of functional connectivity estimates and may enhance prediction of cognitive abilities (Elliott et al., 2019). We took two complementary approaches to examining this brain-behavior relationship. First, we took a hypothesis-driven approach to test whether functional connectivity of the DMN-C subnetwork was specifically related to individual differences in narrative recall. We complemented these analyses with a data-driven approach using connectome-based predictive modeling (Shen et al., 2017) to determine if there were any patterns of whole-brain connectivity that were predictive of narrative recall ability.

## 2 Methods

To answer our research questions, we analyzed data from the Cambridge Center for Aging and Neuroscience (Cam-CAN) repository (Shafto et al., 2014;Taylor et al., 2017). The Cam-CAN repository is a large-scale, cross-sectional, openly available cognitive neuroscience dataset collected by the University of Cambridge. Below we summarize the key characteristics of the Cam-CAN dataset, focusing specifically on the subsets of the dataset that were utilized in the present report. Our analysis plan was preregistered on the Open Science Framework:https://osf.io/9xcu3/?view_only=1ac6856b773249cfa0767dd3d005a9ae.

### 2.1 Participants

Participants included 243 participants between the ages of 18 and 50 years sampled from the original set of 653 Cam-CAN participants who had data available at the time of our access. Participants in the original set were equally sampled from each decile of age from 18 to 87 years of age with approximately equal number of men and women in each decile. They were required to be cognitively healthy, to not have a serious psychiatric condition, to have met hearing and English language requirements for experiment participation, and to be eligible for MRI scanning (Shafto et al., 2014). Informed consent was obtained from all participants included in the study, as described inShafto et al. (2014). Of the 653 available subjects, 7 were missing at least one of the 5 MRI scans (see*MRI Data*), 1 was missing data from logical memory subtest of the Wechsler Memory Scale, 19 were missing Cattell Fluid Intelligence scores, and 1 subject was missing ACE-R data (see*Behavioral Data*). To deal with missing data, we took a listwise deletion approach such that if a participant was missing any of the variables of interest, they were removed from further analysis. Participants were excluded from the current set of analyses if they met the following exclusion criteria. First, participants were excluded from the analysis if they had two or more functional runs that had a mean framewise displacement greater than 0.3 mm, to mitigate the effects of motion on functional connectivity estimates (n = 217). Second, participants were excluded from the analysis if they were older than 50 years of age to mitigate the influence of advanced aging on our results (n = 185). After applying these additional exclusion criteria, we were left with 243 subjects to analyze. These 243 subjects were on average 36.28 years old (median = 36.33 years, SD = 8.42) and 122 self-reported as female and 121 as male.

### 2.2 MRI data

The subset of the Cam-CAN dataset analyzed in the present report contained a single high-resolution T1-weighted anatomical scan, three functional scans, and a single field map to correct for magnetic field inhomogeneities. The three functional scans included a movie-watching scan, a resting-state scan, and a sensorimotor scan. The anatomical and field map images were used during the preprocessing of the functional scans. The three functional scans were used to estimate each subject’s intrinsic functional connectome (see*Functional Connectivity*). The anatomical scan was acquired using a Magnetization Prepared RApid Gradient Echo (MPRAGE) sequence with the following parameters: repetition time (TR) = 2250 ms; echo time (TE) = 2.99 ms; inversion time (TI) = 900 ms; flip angle = 9 degrees; field of view (FOV) = 256 mm × 240 mm × 192 mm; voxel size = 1 mm isotropic; GRAPPA acceleration factor = 2; acquisition time of 4 minutes and 32 seconds. The movie-watching scan involved participants watching an edited version of Alfred Hitchcock’s movie “Bang, You’re Dead”. A total of 193 volumes were acquired using a multiecho, T2*-weighted EPI sequence (TR = 2470 milliseconds, five echoes [TE = 9.4 milliseconds, 21.2 milliseconds, 33 milliseconds, 45 milliseconds, 57 milliseconds], flip angle = 78 degrees, 32 axial slices of thickness of 3.7 mm with an interslice gap of 20%, FOV = 192 mm × 192 mm, voxel size = 3 mm × 3 mm × 4.44 mm) with an acquisition time of 8 minutes and 13 seconds. The resting-state scan involved participants resting in the scanner with their eyes closed. During the sensorimotor scan, participants were presented with visual checkerboards and auditory tones, either in isolation or simultaneously. They were instructed to respond with a button press when they were presented with any stimuli (either visual, auditory, or both visual and auditory). The resting-state and sensorimotor scans had the same scanning parameters: a total of 261 volumes were acquired, each containing 32 axial slices acquired in descending order, slice thickness of 3.7 mm with an interslice gap of 20%; TR = 1970 ms; TE = 30 ms; flip angle = 78 degrees; FOV = 192 mm × 192 mm; voxel size = 3 mm × 3 mm × 4.44 mm) and an acquisition time of 8 minutes and 40 seconds. The field map consisted of an SPGR gradient-echo sequence with the same parameters as the resting-state and sensorimotor tasks, but with two TEs (5.19 ms and 7.65 ms).

### 2.3 Regions of interest

**Fig. 1.**
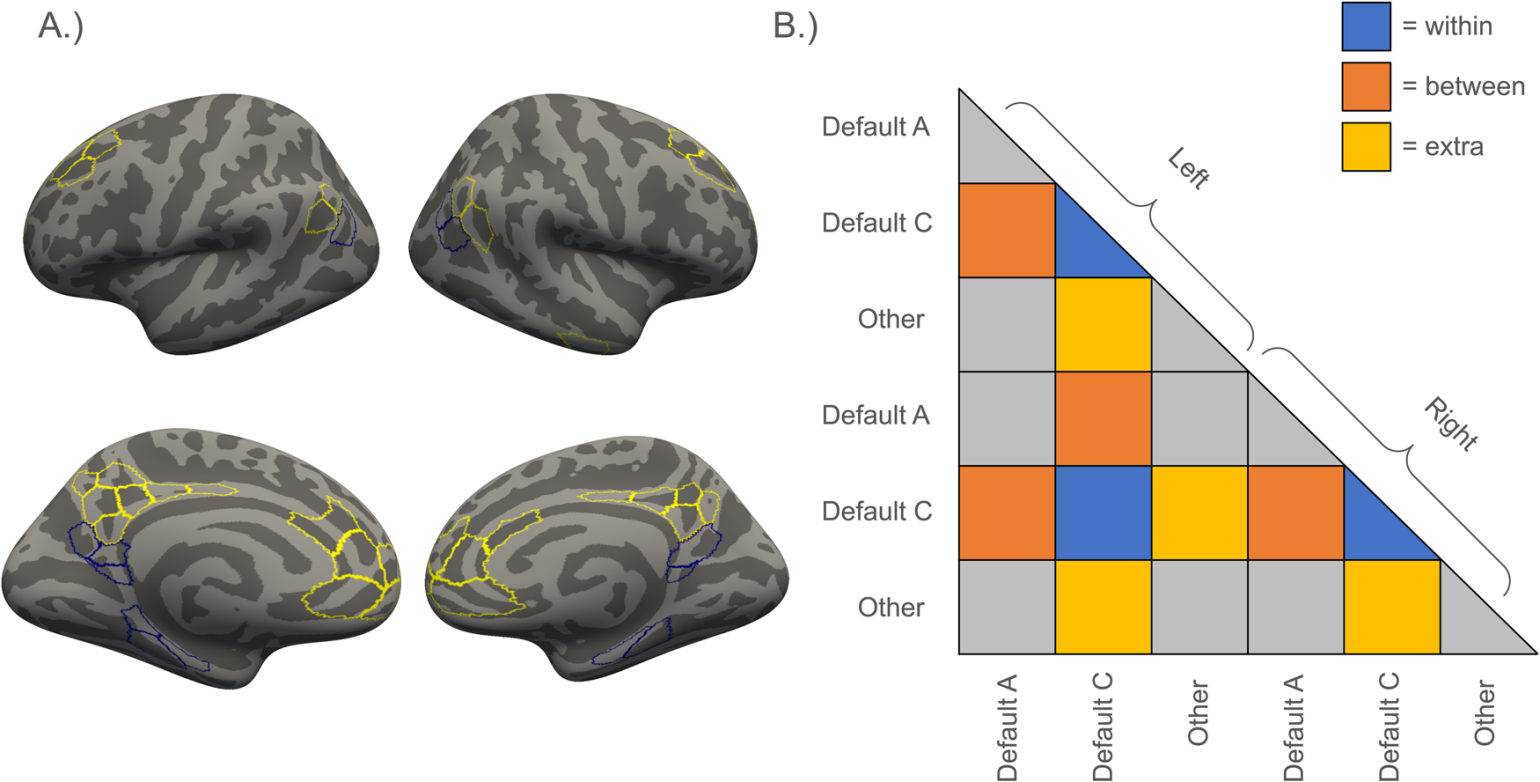
Regions and connections of interest. (A) Regions of interest were based on the 400 parcel 17-network parcellation from the Schaefter atlas (Schaefer et al., 2018). Here we highlight the DMN-C (blue) and DMN-A (yellow) networks from this atlas. This visualization was created in freeview using the*fsaverage*inflated surface template provided in theSchaefer et al. (2018)atlas. (B) The lower triangle of the functional connectome. Connections of interest are highlighted, including “within” connections (blue), “between” connections (orange), and “extra” network connections (yellow) of the DMN-C. Connections of no interest are shown in gray.

Regions of interest (ROIs) were taken from the Schaefer cortical parcellation (Schaefer et al., 2018). Specifically, we used the 400-area resolution, 17-network parcellation. We focused our analyses*a-priori*on three sets of parcels from this atlas: DMN-C regions (number of parcels: left hemisphere = 7, right hemisphere = 6), DMN-A regions (number of parcels: left hemisphere = 18, right hemisphere = 16), and all other regions (number of parcels: left hemisphere = 176, right hemisphere = 178). DMN-C regions consisted of the bilateral parahippocampal gyrus, bilateral retrosplenial cortex, and the bilateral posterior angular gyrus (Fig. 1a, blue regions). DMN-A regions included the bilateral posterior cingulate cortex, the bilateral precuneus, the bilateral medial prefrontal cortex, the bilateral anterior angular gyrus, and a bilateral section of the dorsal prefrontal cortex (Fig. 1a, yellow regions). We focused on regions in the DMN-C and DMN-A networks due to their roles in episodic memory and simulation (Buckner & DiNicola, 2019;Ritchey & Cooper, 2020) and their correspondence with ventral and dorsal subnetworks we have previously identified (Cooper et al., 2021;Kurkela et al., 2022). We note that these were inadvertently mislabeled in our preregistration. To supplement this cortical atlas, we included hippocampal ROIs created using the anatomical delineations fromRitchey et al. (2015). Specifically, we used hippocampal ROIs comprising the hippocampal head, the hippocampal body, and the hippocampal tail from the right and left hemispheres. These six hippocampal ROIs were added to the 400 cortical ROIs to form the functional connectome (i.e., 406 × 406 ROI-to-ROI connectivity matrix).

### 2.4 Behavioral data

Summary statistics for the behavioral and neural variables of interest are presented inTable 1. Behavioral data included performance on the following cognitive assessments: the logical memory subtest from the Wechsler Memory Scale Third UK edition (The Psychological Corporation, 1997), the Cam-CAN emotional memory task (Taylor et al., 2017), the Addenbrook Cognitive Examination-Revised (variable name: ACER), and the Cattell Test of Fluid Intelligence (variable name: Cattell). The logical memory subtest from Wechsler Memory Scale involved having participants read two short passages and subsequently verbally recall as many story details as possible at two different time points: first immediately after reading the short passages and then again after a ~20 minutes delay. Verbal recalls were scored for the number of story details correctly recalled at each point in time. The number of story details recalled at both points in time was averaged together to form a narrative recall score, which serves as our primary measure of memory ability and primary dependent variable. The emotional memory task included recall of object-scene associations (seeTaylor et al., 2017for a description of this task), and the total number of detailed scene recalls was incorporated here as an additional measure of memory ability. Narrative recall and emotional memory scores were standardized across the sample and averaged to create a composite measure of memory ability. Analyses additionally incorporated two general measures of cognitive function. The ACER is a standardized cognitive battery originally designed for dementia screening. The battery is designed to test participants’ ability in five different cognitive domains, including attention, memory, fluency, language, and visual-spatial. The total score on this battery was used as an index of general cognitive function. The Cattell Test of Fluid Intelligence is a timed pen and paper task where participants are required to solve a series of nonverbal puzzles. Here we use the total score on this task as a general index of fluid intelligence.

**Table 1.** Data summary.

						Correlations									
Variable	mean	min	max	sd	n	mem-narr	mem-emo	Age	Sex	Cattell	ACER	fd	within	between	extra
mem-narr	15.28	2.50	23	3.50	243										
mem-emo	52.98	4	105	23.98	120 ^b^	0.44									
Age	36.28	18.50	49.83	8.42	243	-0.13	-0.32								
Sex ^a^	0.50	0	1		243	0.14	0.10	-0.07							
Cattell	36.35	22	44	4.23	235 ^c^	0.35	0.30	-0.25	-0.19						
ACER	96.54	74	100	3.63	243	0.40	0.31	0.12	0.03	0.39					
fd	0.14	0.05	0.25	0.04	243	-0.10	-0.11	0.23	0.02	-0.22	-0.14				
within	0.31	0.17	0.50	0.06	243	0.02	0.09	-0.21	0.02	0.09	0.04	-0.23			
between	0.15	0.03	0.26	0.05	243	0.04	-0.08	-0.01	0.09	-0.06	0.03	-0.10	0.53		
extra	-0.02	-0.06	0.02	0.01	243	-0.09	-0.16	0.14	0.02	-0.06	-0.07	0.14	-0.55	-0.63	
hipp	0.00	-0.03	0.04	0.01	243	-0.10	-0.22	-0.02	-0.18	0.01	-0.03	-0.07	0.02	-0.19	0.28

Statistical summary of data analyzed for the current report.*mem-narr *= number of story details recalled in the logical memory subtest of the Wechsler Memory Scale—average of immediate and delayed,*mem-emo *= total number of detailed scene recalls in the Cam-CAN emotional memory task,*Cattell*= total score on the Cattell Test of Fluid Intelligence,*ACER*= total score on Addenbrook’s Cognitive Evaluation Revised (ACE-R),*fd*= mean framewise displacement,*within*= average functional connectivity estimate (Pearson’s correlation*r*) of DMN-C—DMN-C connections,*between*= average functional connectivity estimate (Pearson’s correlation*r*) of DMN-C–DMN-A connections,*extra*= average functional connectivity estimate (Pearson’s correlation*r*) for DMN-C–rest of the brain connections, n = number of complete observations. Correlations were calculated using all available complete pairs of data. All values are rounded to two decimal places where appropriate.^a^Sex was coded such that Female = 1, Male = 0.^b^Half of subjects completed Cam-CAN’s emotional memory task (seeShafto et al., 2014).^c^8 Subjects Missing Cattell Scores.

### 2.5 Analysis

#### 2.5.1 MRI quality control

The quality of the functional MRI data was assessed using the*MRIQC*software package (Esteban et al., 2017). Quality reports generated from this software package were visually inspected for scanner artifacts and motion-related corruption. All scans that had a mean framewise displacement greater than 0.3 mm were excluded from further analysis. If subjects had two or more scans with a mean framewise displacement greater than 0.3 mm, then the subject was excluded from further analysis (see*Participants*). This conservative approach was adopted in order to limit the effects of head motion on functional connectivity measures (seeCooper et al., 2021for a similar exclusion criterion;Power et al., 2012,^2014^).

#### 2.5.2 MRI preprocessing

MRI data were preprocessed using*fMRIPrep*20.2.0 (Esteban et al., 2019). Processing steps for the T1w images included correction for intensity nonuniformity, skull stripping, brain tissue segmentation, and volume-based spatial normalization to the ICBM 152 Nonlinear Asymmetrical template version 2009c. Processing steps for the three BOLD runs included slice-timing correction, realignment, using the field map to optimize coregistration of the functional images to the anatomical reference image, normalization of the BOLD images to the ICBM 152 Nonlinear Asymmetrical template version 2009c, and the calculation of confounding time series including basic six head-motion parameters (x,y,z translation; pitch, roll, yaw rotation), temporal derivatives, and the quadratic terms of the head-motion parameters, noise components from a principal components analysis based denoising routine (*CompCorr*), framewise displacement, and DVARS. For a much more detailed description of the processing pipeline, please refer to theSupplemental Materialswhich contains the recommended*fMRIPrep*boilerplate.

#### 2.5.3 Functional connectivity

Functional connectivity analyses were performed using the*CONN*toolbox (Whitfield-Gabrieli & Nieto-Castanon, 2012). Confounds removed from each voxel’s time series included six head-motion parameters and their temporal derivatives, up to the first six*aCompCor*components from a combined WM and CSF mask, framewise displacement, and the global signal^1^as calculated by*fMRIPrep*. Additional spike regressors were included for any time points that exceeded an FD of 0.6 mm and/or a standardized DVARS of 2. After regression of motion confounds, BOLD data were band-pass filtered with a high-pass filter of 0.008 Hz and a low-pass filter of 0.1 Hz. No additional modeling was performed for the movie-watching and the resting-state data. For the sensorimotor task, task-related activations were regressed out of the time series prior to calculation of functional connectivity. Specifically, task-related activation was modeled in the sensorimotor task by convolving stick functions placed at stimulus onsets with SPM12’s hemodynamic response function. ROI-to-ROI functional connectivity was calculated using the Pearson's correlation coefficient after denoising and task modeling. The ROI-to-ROI intrinsic functional connectivity estimates were created by averaging functional connectomes calculated from all available functional scans, resulting in a measure of “generalized” functional connectivity intrinsic to each individual (Elliott et al., 2019). The resulting ROI-to-ROI intrinsic functional connectomes were then summarized to three terms to test our research hypotheses.*Within*subnetwork connectivity was operationalized as the average of all functional connections among DMN-C nodes in theSchaefer et al. (2018)atlas,*between*subnetwork connectivity as the average connectivity between all DMN-C and DMN-A nodes, and*extra*network connectivity as the average of all connections between the DMN-C nodes and all other nodes not contained in DMN-C or DMN-A. SeeFigure 1bfor an illustration. Although averaging across the three scan types has the advantage of increasing the amount of fMRI data used to estimate functional connectivity and increasing the reliability of the resulting functional connectivity estimates (Elliott et al., 2019), it may obscure task-specific relationships between functional connectivity and behavior, particularly in the context of tasks that vary considerably in their content and demands. Thus, we additionally conducted exploratory analyses in which functional connectivity relationships were analyzed separately for each of the three scan types (resting-state, movie-watching, and sensorimotor task).

#### 2.5.4 Statistical modeling

All statistical models, tables, and figures were created using R (R Core Team, 2022). Linear regression models were used to estimate the relationship between summary measures of functional connectivity and memory ability, on their own and then including covariates for age, sex, average framewise displacement, fluid intelligence, and cognitive function as defined under*Behavioral Data*. Standardized beta coefficients are reported. Summary tables for the linear regressions were created using a combination of the R packages*gtsummary*(Sjoberg et al., 2021) and*flextable*(Gohel & Skintzos, 2023). All figures were created using the R package*ggplot2*(Wickham, 2016).

#### 2.5.5 Connectome-based predictive modeling

To complement the hypothesis-driven approach described above, the current report used connectomic-based predictive modeling (CBPM;Shen et al., 2017). CBPM involves calculating a functional connectome for each individual and determining which connections are statistically related to a behavioral variable of interest. All connections that are related to behavior above some arbitrarily defined threshold (e.g., at*p*< 0.01) are then summarized by separating out connections with a significant positive correlation with behavior from those that have a significant negative correlation with behavior. The connections in the connectome with significant positive and negative correlations with behavior are then summed into separate positive and negative terms for use in a linear regression predicting behavior. The analysis method then estimates a linear regression using these summed positive and negative connection terms to predict the behavior of a left-out participant in a leave-one-out cross-validation procedure. Successful prediction of a behavioral variable using the functional connectome is then determined by correlating the predicted behavioral scores for each participant with the observed behavioral scores with statistical significance determined using a null permutation procedure that reruns the entire analysis a given number of times, each time randomly pairing connectomes with behavioral scores. All CBPM analyses were performed using a connection selection threshold of*p*< 0.01 and the null distribution was formed from 100 null simulations. The results from alternative connection selection thresholds are reported in theSupplemental Materials. Statistical significance of the CBPM analysis was determined by estimating the proportion of null simulations that resulted in better predictive performance than the actual analysis. All CBPM analyses were performed using modified analysis code published byShen et al. (2017). To control for nuisance variables, the CBPM analysis code published by Shen and colleagues was modified to use the MATLAB function*partialcorr*when selecting connections used for prediction. To determine which connections in the connectome the models were relying on to make their predictions, we also performed computational lesion analyses. In a computational lesion analysis, a predictive model is iteratively fit while excluding a set of features. If model performance is hindered by the removal of a set of features, then it is inferred that the model is relying on these features to make successful out-of-sample predictions.

## 3 Results

### 3.1 Functional connectivity within the default-C subnetwork

**Fig. 2.**
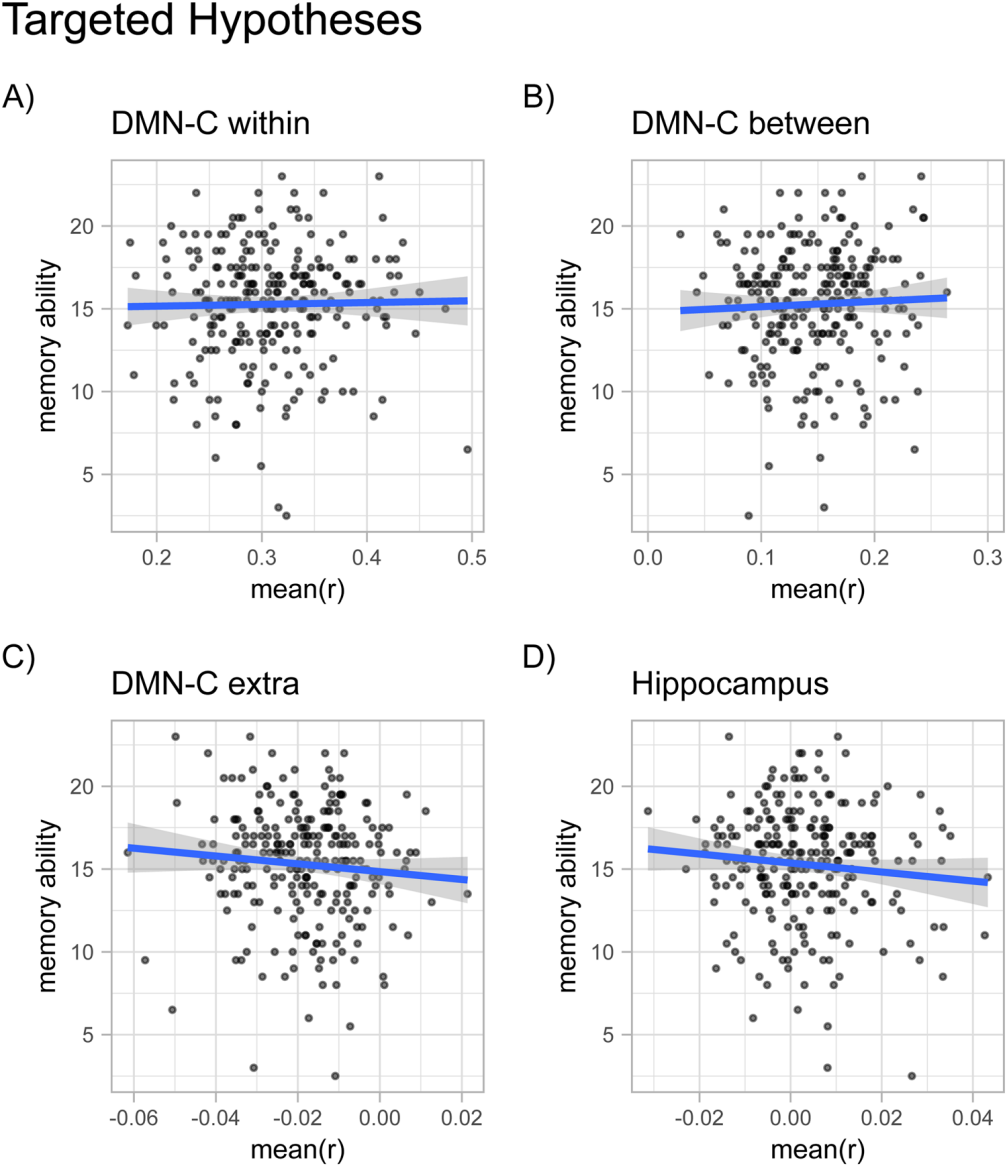
Relationships between memory ability and intrinsic functional connectivity among memory networks. Scatter plots and best fit linear regression line of narrative recall performance on average intrinsic connection strength (A) among DMN-C regions, (B) between DMN-C and DMN-A regions, (C) DMN-C and all other regions, and (D) the Hippocampus and all regions. There was little evidence to suggest that the strength of these connections was predictive of individual differences in memory ability.

Average intrinsic functional connectivity among DMN-C regions was not related to narrative recall (*β*= 0.06,*SE*= 0.23,*t*(241) = 0.28,*p*= 0.78;Fig. 2a). This pattern did not change after controlling for age, sex, and average framewise displacement (*β*= -0.10,*SE*= 0.23,*t*(238) = -0.42,*p*= 0.68) or after also controlling for fluid intelligence and cognitive function (*β*= -0.06,*SE*= 0.21,*t*(228) = -0.28,*p*= 0.78).

### 3.2 Functional connectivity between the default C and default A subnetworks

Average between subnetwork connectivity did not predict narrative recall on its own (*β*= 0.15,*SE*= 0.23,*t*(241) = 0.66,*p*= 0.51;Fig. 2b), nor did it predict narrative recall when controlling for age, sex, and average framewise displacement (*β*= 0.07,*SE*= 0.22,*t*(238) = 0.33,*p*= 0.74), nor when additionally controlling for fluid intelligence and cognitive function (*β*= 0.19,*SE*= 0.20,*t*(228) = 0.96,*p*= 0.34).

### 3.3 Functional connectivity between the default C subnetwork and all other brain regions

The average strength of functional connections from DMN-C regions to regions in networks other than the DMN-A and DMN-C did not predict narrative recall on its own (*β*= -0.31,*SE*= 0.22,*t*(241) = -1.39,*p*= 0.17;Fig. 2c), when controlling for age, sex, and average framewise displacement (*β*= -0.24,*SE*= 0.23,*t*(238) = -1.05,*p*= 0.29), nor when additionally controlling for fluid intelligence and cognitive function (*β*= -0.25,*SE*= 0.20,*t*(228) = -1.23,*p*= 0.22).

### 3.4 Functional connectivity of the hippocampus

Although not part of our preregistered set of analyses, we decided to look at the relationship between narrative recall and intrinsic functional connectivity of the hippocampus given prior research linking this region to individual differences in memory (L. Wang, LaViolette, et al., 2010;L. Wang, Negreira, et al., 2010;Touroutoglou et al., 2015). We set about testing this in a similar manner to our preregistered set of analyses. We found that the average strength of all hippocampal connections was not a significant predictor of narrative recall on its own (*β*= -0.34,*SE*= 0.22,*t*(241) = -1.5,*p*= 0.13;Fig. 2d). This result held when we statistically controlled for age, sex, and average framewise displacement (*β*= -0.29,*SE*= 0.23,*t*(238) = -1.28,*p*= 0.2), and when we further controlled for fluid intelligence and cognitive function (*β*= -0.26,*SE*= 0.20,*t*(228) = -1.3,*p*= 0.2). We note here that the inclusion of global signal regression in our analysis pipeline influenced this result in that exclusion of this step resulted in a significant negative association between hippocampal connectivity and narrative recall (seeSupplemental Materialsfor further discussion).

### 3.5 Task-specific relationships between functional connectivity and memory ability

To explore whether there might be task-specific relationships between functional connectivity and memory, we reran the above analyses separately for each scan type (resting-state, movie-watching, and sensorimotor task). There were no significant relationships between any of the task-specific functional connectivity measures and narrative recall, all*p*’s > 0.05.

We additionally sought to determine whether this pattern of null results was specific to the relationship between functional connectivity and narrative recall performance. To do so, we calculated a composite memory ability score incorporating the narrative recall measure as well as performance on an emotional memory task collected in a subset of our sample (N = 120). This composite measure was included as the dependent variable in the regression models analogous to those reported above. The results were largely similar to those reported for the narrative recall analyses above, with no significant relationships between composite memory ability and functional connectivity among DMN regions. One exception was that in these analyses, there was a significant negative relationship between hippocampal functional connectivity and composite memory ability,*β*= -0.21,*SE*= 0.08,*t*(118) = -2.73,*p*= 0.007, which held up after controlling for age, sex, framewise displacement, fluid intelligence, and cognitive function,*β*= -0.17,*SE*= 0.07,*t*(110) = -2.53,*p*= 0.013. We note that a similar numerical pattern was observed in the narrative recall analyses, suggesting that these results reflect a quantitative but not a qualitative shift in the brain-behavior relationship.

### 3.6 Connectome-based predictive modeling

In a planned exploratory analysis, we ran a CBPM analysis to see if we could predict narrative recall using the entire intrinsic functional connectome. The grand mean functional connectome for our sample is reported in theSupplemental Materials. The first analysis—where we used the entire 406 × 406 intrinsic functional connectome to predict narrative recall—resulted in a significant correlation between observed and predicted narrative recall scores (*r*_{observed, predicted}_= 0.164,*p*= 0.027)—suggesting that there is a multivariate pattern within the broader functional connectome that is predictive of individual differences in narrative recall.

To ensure the robustness of this result, we first reran the CBPM analysis while controlling for nuisance variables when selecting connections to be used for predicting left-out subjects (using the MATLAB function*partialcorr*, seeShen et al., 2017). Specifically, we saw that the predictive performance of the intrinsic connectome held when controlling for age, self-reported sex, and average framewise displacement (*r*_{observed, predicted}_= 0.1498,*p*< 0.01). All subsequent CBPM analyses were performed controlling for age, self-reported sex, and average framewise displacement. We next tested whether this result held when selecting different connection selection thresholds. In line with previous reports that the CBPM method is robust to connection selection threshold (Finn et al., 2015;Jangraw et al., 2018;Shen et al., 2017), rerunning the CBPM analysis using connection selection thresholds of*p *= [0.001, 0.005, 0.01, 0.5, 0.1] had similar outcomes (Supplemental Materials). Finally, we examined whether these results were affected by the inclusion of global signal regression in our processing pipeline (seeSupplemental Materials). Removing the global signal regression resulted in nonsignificant prediction performance,*r*_{observed, predicted}_= 0.081,*p*= 0.099. Together, these findings gave us confidence that our CBPM results were robust to most analytic decisions, while it appears that including global signal regression improved predictive performance.

We then examined how combining functional connectomes from different tasks influenced our model. Previous research suggests that functional connectivity calculated by collapsing across resting-state and task scans increases the reliability and thus the predictive utility of individuals’ functional connectomes (Elliott et al., 2019). To confirm that this was the case in our data, we reran the CBPM analysis on functional connectomes calculated using the movie-watching, resting-state, and sensorimotor task scans separately. Models built using functional connectomes from each task individually underperformed compared with the model built using the combined intrinsic functional connectome. Among the three tasks, the model built using functional connectomes from the movie-watching scan was the only one able to predict narrative recall better than chance. Interestingly, models built using resting-state data performed particularly poorly, with almost no relationship between observed and predicted narrative recall scores in out-of-sample data (Supplemental Materials). These results support previous reports that suggested that functional connectivity estimates calculated across task and resting-state scans outperform those from short resting-state scans alone (Elliott et al., 2019).

**Fig. 3.**
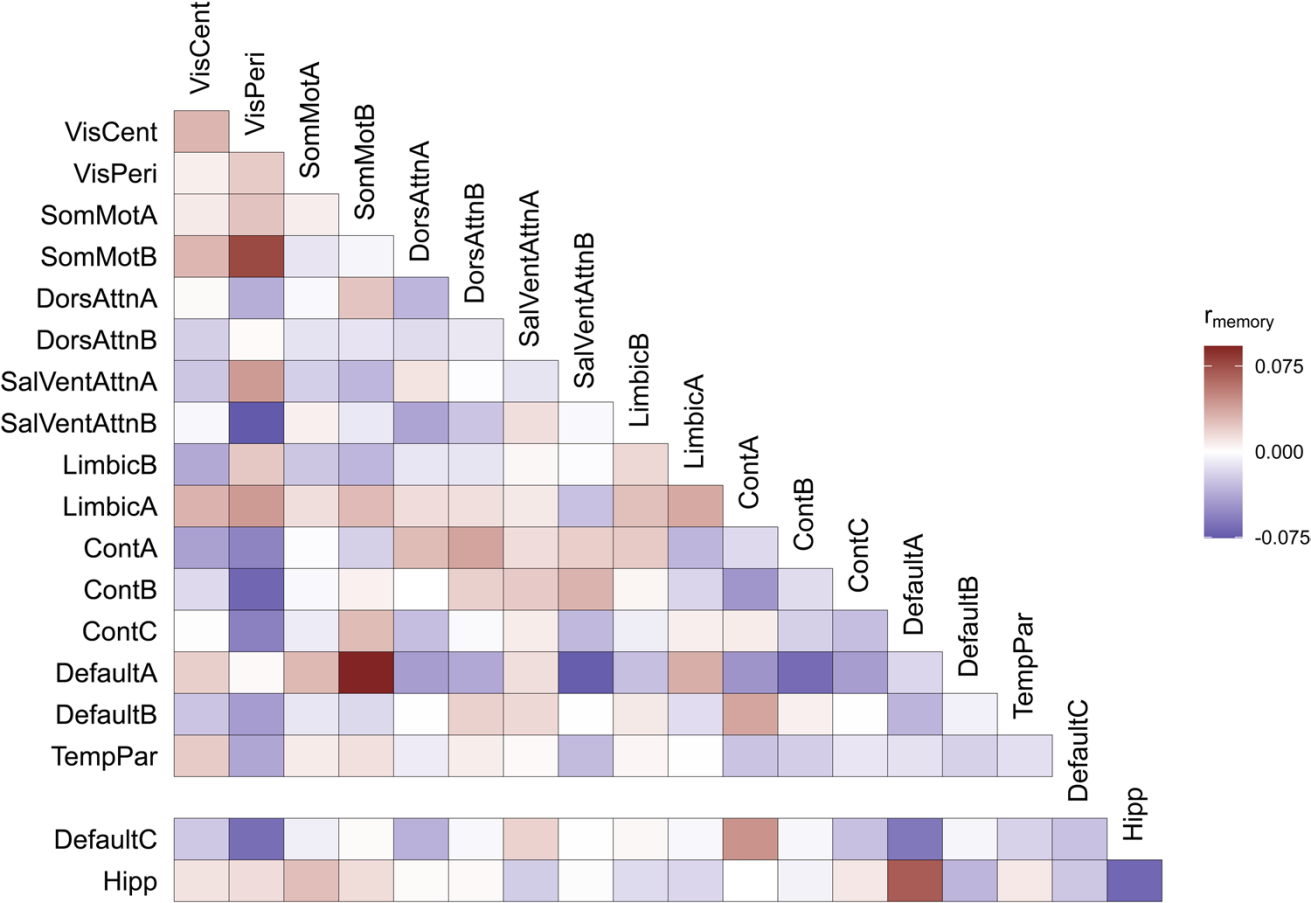
Evaluating Feature Importance. The partial correlation of connection strength with narrative recall scores after controlling for age, sex, and average in-scanner motion. DMN-C and hippocampal connections are offset to highlight these memory-related regions. SeeYeo et al. (2011)andSchaefer et al. (2018)for descriptions of each network.

To identify which networks were driving model performance, we ran a computational lesion analysis. In the computational lesion analysis, we reran our CBPM analysis excluding each network from the analysis in turn. Network importance in this computational lesion analysis was determined via a significant drop in model performance with the exclusion of a network and its connections. The results of our computational lesion analyses are reported inTable 2. Exclusion of typical memory-related brain regions (i.e., DMN-C regions and the hippocampus) had little impact on model performance. Among nonmemory-related networks, the Somatomotor Network B was the only network whose exclusion led to a significant drop in model performance. To visualize the specific connections related to narrative recall,Figure 3depicts the partial correlation between intrinsic functional connectivity and narrative recall scores for every connection in the functional connectome, averaged within each network. We also calculated the number and proportion of connections between each of our networks of interest that exceeded our connection selection threshold of*p*< 0.01 (seeSupplemental Materials), as these constitute the connections that actually went into the CBPM analysis. These visualizations reveal a pattern of increases and decreases throughout the connectome associated with individual differences in narrative recall. Taken all together, these results suggest that memory ability-related information was largely contained within connections of nonmemory-related brain regions.

**Table 2.** Computational lesion analysis.

	*r*	*p* (null_sim)
Nonmemory Networks		
VisCent	0.156	0.000
VisPeri	0.152	0.010
SomMotA	0.169	0.000
SomMotB	-0.012	0.386
DorsAttnA	0.175	0.000
DorsAttnB	0.143	0.000
SalVentAttnA	0.168	0.000
SalVentAttnB	0.165	0.000
LimbicB	0.130	0.010
LimbicA	0.143	0.000
ContA	0.139	0.010
ContB	0.155	0.000
ContC	0.148	0.000
DefaultA	0.169	0.000
DefaultB	0.094	0.040
TempPar	0.133	0.010
Memory Networks		
DefaultC	0.166	0.000
Hipp	0.154	0.000

Excluding networks of regions traditionally related to episodic memory ability—i.e., the default mode network C and the Hippocampus—has very little impact on model performance. Among nonmemory networks, only excluding Somatomotor Network B regions from the analysis resulted in a significant drop in model performance.*r*= Pearson’s correlation between observed and predicted narrative recall scores,*p*= proportion of null simulations that were more extreme than observed. SeeYeo et al. (2011)andSchaefer et al. (2018)for descriptions of each network.

## 4 Discussion

The current report examined how individual differences in episodic memory ability are related to intrinsic functional connectivity, focusing specifically on regions in the default mode network (DMN-C) and the hippocampus that have been strongly implicated in episodic memory (Ritchey & Cooper, 2020). Across a sample of 243 individuals, we found little evidence that the strength of DMN-C and hippocampal functional connections was related to narrative recall performance. We did find evidence, however, that connectome-wide patterns of functional connectivity could predict individual differences in narrative recall. These predictive models were robust to analytic decisions and remained significant after controlling for age, self-reported sex, and average in-scanner movement. Probing these predictive models further revealed that superior narrative recall performance was related to functional connectivity of brain regions not typically related to memory ability—specifically, to somatomotor regions. Together, these results suggest that there is limited evidence connecting individual differences in memory to measures of intrinsic functional connectivity among regions typically associated with memory function.

The null results reported here were contrary to our*a priori*hypotheses, and they contrast with previous reports linking changes in intrinsic functional connectivity of default mode network (Sneve et al., 2017;van Buuren et al., 2019) and hippocampal regions (L. Wang, LaViolette, et al., 2010;L. Wang, Negreira, et al., 2010;Setton, Mwilambwe-Tshilobo, et al., 2022;Touroutoglou et al., 2015) to episodic memory ability. One possible explanation for this set of results is that the strength of intrinsic functional connections might not be reliably related to episodic memory ability, despite its relationship to other measures of cognitive performance. Measures of intrinsic functional connectivity, often obtained from resting-state scans that do not include an explicit cognitive task, have been widely used to study individual differences in cognition. Strong evidence has accumulated over the years to suggest that these measures are related to behavioral phenotypes. Studies of the resting state have found that there is a normative pattern of functional connections in the brain, such that brain regions form stable networks (between 7 and 17,Yeo et al., 2011). Recent work suggests that the majority of variability in the strength of these connections is attributable to stable individual differences away from this group-level pattern (as opposed to variation attributable to cognitive task or day-to-day variation; (Gratton et al., 2018). Furthermore, the strength of intrinsic connections has been shown to be predictive of a number of different behavioral phenotypes including neuroticism and extraversion (Hsu et al., 2018), trait-level anxiety (Z. Wang et al., 2021), fluid intelligence (Finn et al., 2015), creativity (Beaty et al., 2018), sustained attention (Rosenberg et al., 2016), and working memory ability (Avery et al., 2020). Patterns within the intrinsic functional connectome are so identifiable that they can be used to identify an individual from a group, acting as a sort of “brain fingerprint” (Finn et al., 2015). Thus, it seemed reasonable to hypothesize that individual differences in intrinsic functional connectivity would be related to memory ability.

Other work critiques this line of research, however, suggesting that “intrinsic” functional connectivity calculated in the resting state is less useful for predicting individual differences compared with “active” functional connectivity calculated while participants complete cognitive tasks (Greene et al., 2018,^2020^;Lin et al., 2021), especially tasks that are relevant to the predicted cognitive ability (Zhao et al., 2023). An analogy would be examining two cars and trying to determine which one is the better race car—you may not be able to tell the difference between an expensive race car and a Toyota Civic by looking under the hood when they are sitting in a garage, but you may be able to tell the difference when you examine how their mechanical parts perform when engaged in a race. The results of one of our supplemental analyses support this idea, with out-of-sample performance being significantly worse using the resting-state scan compared with the movie-watching or sensorimotor task data (Supplemental Materials). A closely related critique also questions the use of resting-state networks for understanding how the brain supports cognition. This critique specifically puts forth the idea that the most important unit of analysis when relating brain measures to cognition is not networks identified via low-frequency coactivation during rest, but networks identified via high-frequency, transient coupling while participants complete highly controlled cognitive tasks (Cabeza & Moscovitch, 2013;Davis et al., 2017;Moscovitch et al., 2016). These “process-specific alliances” often straddle resting-state network definitions, and in contrast to resting-state networks, they are thought to be able to flexibly assemble and disassemble depending on task demands. For example, interactions between the hippocampus and the left ventrolateral prefrontal cortex are thought to support successful episodic encoding (e.g.,Wing et al., 2013), despite the fact that hippocampus is typically a member of the default mode network and left ventrolateral prefrontal cortex is not (Davis et al., 2017). Thus, it could be the case that individual differences in behavior manifest themselves in individual differences in the strength of these high-frequency and cognition-specific alliances and not in strength of low-frequency resting-state networks. Here, we combined data from resting-state and task scans in order to estimate a “generalized” measure of functional connectivity (Elliott et al., 2019), which is thought to be a more reliable measure than those derived from short resting-state scans alone, but will necessarily obscure task-specific variation. When considering the scans separately, we found largely similar results across the different types of scans. However, there was evidence for significant connectome-based prediction only for the movie-watching scan, which is notable because processing event narratives in the context of movie watching may be more relevant to the kinds of processes that support performance on narrative recall tasks (Jääskeläinen et al., 2021). Thus, although we did not find a reliable relationship between memory ability and intrinsic measures of functional connectivity in memory-related brain regions, we hypothesize that this relationship may be apparent when measuring brain activity while participants complete a memory-related task, or when incorporating brain measures that isolate a particular pattern of activity related to memory function (e.g.,Richter et al., 2023).

In our data-driven analyses, we found that a multivariate brain-wide pattern of functional connectivity could predict individual differences in narrative recall reliably out-of-sample. Our predictive analyses suggest that the brains of superior rememberers are characterized, in part, by decoupling of somatomotor regions and DMN-A regions and increased communication between somatomotor regions and visual processing regions. We speculate that the involvement of somatomotor regions in predicting memory ability might be explained by variation in goal-directed attention in the sensorimotor task, as successful attention in this task might be related to attentional factors contributing to individual differences in memory (Unsworth, 2019). The decoupling of default and sensory regions is in line with previous results that likewise showed evidence that the superior rememberers were characterized by decoupling between default mode and sensory networks (Sneve et al., 2017). Other studies, however, have shown that general increases in task-related DMN connectivity are related to episodic memory ability (King et al., 2015) and that lower MTL-DMN and higher DMN-frontoparietal control network connections are related to memory ability (van Buuren et al., 2019). A recent, well-powered study identified multivariate patterns of associations between individual differences in autobiographical memory and functional connectivity among DMN regions, including the temporal pole and hippocampus, although these relationships were largely outside of the neocortical regions most commonly associated with episodic memory (Setton, Mwilambwe-Tshilobo, et al., 2022). What is clear from the results presented here and the literature to date is that information about memory ability in healthy young adults is likely contained within the functional connectome beyond brain networks classically linked to episodic remembering (Lin et al., 2021). The exact pattern of whole-brain connectivity that predicts memory ability, however, remains inconsistent from study to study. As such, we refrain from drawing strong conclusions about the specific connections related to memory in our connectome-based predictive modeling analyses.

An important factor that may have influenced our results is our choice of memory measure. Here, we used a standard neuropsychological measure of memory that assessed an individual’s ability to recall the details of a short narrative—the logical memory subtest of the Wechsler Memory Scale (The Psychological Corporation, 1997). The logical memory subtest of the Wechsler Memory Scale was chosen because it was available for the largest number of subjects in the Cam-CAN dataset, compared with other included memory measures, and this measure has been previously shown to correlate with individual differences in brain activity in response to event boundaries during the Cam-CAN movie-watching scan (Reagh et al., 2020). Narrative recall has also been linked specifically to the function of the DMN, such that there is increased activity and functional integration among DMN regions during recall of event narratives (Lee et al., 2020;Ritchey & Cooper, 2020). Reliance on a single memory measure to capture an individual's memory ability, however, may be problematic given previous research suggesting that individuals have aptitudes for different types of memory tasks (Unsworth, 2019). We speculate that differences in how memory ability is operationalized could explain the mixed state of the literature—all of the previous research relating individual differences in memory ability and functional connectivity have taken a unique approach to operationalizing memory ability (King et al., 2015;Lin et al., 2021;Sneve et al., 2017;van Buuren et al., 2019).King et al. (2015)andSneve et al. (2017)relied on performance in source memory recognition tasks;Lin et al. (2021)relied on performance on a remember/know/new recognition memory paradigm;van Burren et al. (2019)relied on performance on an object-location memory task;Setton, Mwilambwe-Tshilobo, et al. (2022)used the number of internal details generated during an autobiographical memory interview. The brain areas supporting retrieval depend in part on the content, modality, and format of memory tasks (Diana et al., 2007), consistent with the idea that memory performance is supported by a combination of task-specific and task-general processes (Rugg & Vilberg, 2013). Such differences in the content and format of memory tasks may explain why there has not been one consistent neural signature associated with performance. Here, we attempted to test the generalizability of our results by creating a composite measure of memory ability in a subset of our sample, again finding little evidence for a relationship between DMN functional connectivity and memory ability. In this set of analyses, hippocampal functional connectivity became significantly associated with individual differences in composite memory ability, though surprisingly, this relationship was in the negative direction. That is, individuals with higher composite memory scores had lower intrinsic functional connectivity between the hippocampus and all other regions, contrasting with prior work (L. Wang, LaViolette, et al., 2010;L. Wang, Negreira, et al., 2010;Setton, Mwilambwe-Tshilobo, et al., 2022;Touroutoglou et al., 2015; but seeMatijevic et al., 2022). Due to the unexpectedness of this result and its sensitivity to analysis decisions, we remain cautious in interpreting this finding but consider it a target for further investigation. Future research on individual differences in memory ability should also incorporate multiple memory measures to get an unbiased measure of individuals’ overall memory ability, or alternatively, systematically select and compare memory measures that are likely to correspond with dissociable neural processes (e.g.,Ngo et al., 2021).

While the studies reviewed above have considered individual differences in objective evaluations of participants’ memory, other studies have looked at measures of individuals’ subjective evaluations of their own memory when relating individual differences to brain function (Petrican et al., 2020;Sheldon et al., 2016).Sheldon et al. (2016), for example, examined the relationship between the strength of functional connectivity of the parahippocampal cortex and subjective reports of episodic memory tendencies measured by the Survey of Autobiographical Memory (SAM) (Palombo et al., 2013). The SAM measures participants’ self-reported mnemonic traits, measuring their tendency, for example, to remember specific event and contextual details when recalling events (episodic subscale) versus their tendency to remember facts about oneself, events, or the world that lack contextual detail (semantic subscale). There is some evidence that individual differences in self-reported mnemonic traits measured by the episodic subscale of the SAM are associated with a similar resting-state functional connectivity profile as individual differences are measured using a visual, laboratory-based episodic memory task (Petrican et al., 2020). Future work should consider the relationship between subjective and objective measures of memory function (e.g.,Clark & Maguire, 2020;Cooper & Ritchey, 2022;Setton, Lockrow, et al., 2022) in the context of how these measures relate to individual differences in brain function.

An intriguing future direction is to look at other facets of brain connectivity and how they may be related to episodic memory ability. There are at least three facets of brain connectivity that could theoretically support individual differences in cognition: variability in connectional strength, variability in the spatial localization of brain regions, and variability in large-scale network topology (i.e., large-scale networks have different sets of constituent nodes across subjects;Gordon & Nelson, 2021). The current study, like many of the studies that have come before, focused on how variability of the strength of functional connections related to individual differences in memory ability. Recent work, however, suggests that there are substantial individual differences in the size and topological organization of functional brain areas (Gordon, Laumann, Adeyemo, et al., 2017;Gordon & Nelson, 2021;Kong et al., 2019;Laumann et al., 2015).Gordon and Nelson (2021)display a striking example of this type of idiosyncrasy, where the posterior medial precuneus node of the default mode network for one subject is translated along the cortical surface such that the node wraps around to the lateral side of the brain. Recent work has also identified individual differences in large-scale network topology, such that nodes that are the same across individuals are a part of different large-scale networks (Gordon & Nelson, 2021;Laumann et al., 2015;Seitzman et al., 2019). Such “network variants” appear to occur in specific regions of the brain, particularly in default mode regions, and are observed in some datasets in approximately 33% of individuals (Seitzman et al., 2019). These differences in the spatial topography of functional nodes on the cortical surface also appear to have behavioral relevance. Individuals that have similar spatial network topographies also perform similarly on cognitive tasks, so much so that individual behavior can be predicted out of sample using similarity in brain-wide network topography and network size (Kong et al., 2019). An intriguing possibility is that individual differences in memory ability could be related to the presence or absence of network variants in default mode regions.

In conclusion, we found little evidence in support of a relationship between individual differences in narrative recall and intrinsic functional connectivity among cortical and hippocampal networks commonly associated with episodic memory. We did find a multivariate brain-wide pattern of functional connectivity that was predictive of narrative recall, characterized by decoupling of somatomotor regions from default mode regions. Our findings agree with previous research that suggests that information about memory ability is contained in functional connections among regions outside of those classically linked to episodic memory. The exact nature of this brain connectivity pattern that predicts memory ability remains unclear. We believe this is due to variability in how memory ability is operationalized and the low power of previous studies both in terms of number of subjects collected and in terms of the amount of data collected per subject. Future research on the relationship between individual differences in memory and functional brain networks should incorporate multiple measures to operationalize memory ability unbiased toward a particular task (Unsworth, 2019), collect a large amount of data (Gordon, Laumann, Adeyemo, et al., 2017;Marek et al., 2022), especially task-related data (Greene et al., 2018), and examine additional facets of brain organization that may better capture variability across individuals (Gordon & Nelson, 2021).

## Supplementary Material

Supplementary Material

## Data Availability

The current study reports data that are openly available from the Cambridge Center for Aging Neuroscience (Shafto et al., 2014;Taylor et al., 2017). Custom code used to process and analyze these data is available in the following repository:https://github.com/memobc/paper-CamCanIDs

## Footnotes

1 Global signal regression (GSR) was not included in the pre-registered analysis plan. However, since pre-registration, we have been persuaded by recent publications suggesting that GSR optimizes predicting individual differences in behavior from functional connectivity estimates (Finn & Bandettini, 2021;Li et al., 2019). We report the results without global signal regression in theSupplemental Materials. The conclusions regarding the DMN remain the same across methods, with some differences in the hippocampal and CBPM analyses noted in the Results andSupplemental Materials.
