# Supplementary material for: Intrinsic functional connectivity among memory networks does not predict individual differences in narrative recall
Source: Imaging Neurosci (Camb). 2024 May 20;2:imag-2-00169. doi: 10.1162/imag_a_00169 (PMC12247584; doi:10.1162/imag_a_00169)
Supplement: Supplementary Material [file imag_a_00169-supp.pdf]

Intrinsic functional connectivity among memory networks does not predict individual differences in narrative recall: Supplemental Materials

Kyle Kurkela & Maureen Ritchey

Department of Psychology and Neuroscience, Boston College

Correspondence concerning this article should be addressed to:

Maureen Ritchey
Boston College Department of Psychology and Neuroscience
Chestnut Hill, MA
Email: maureen.ritchey@bc.edu

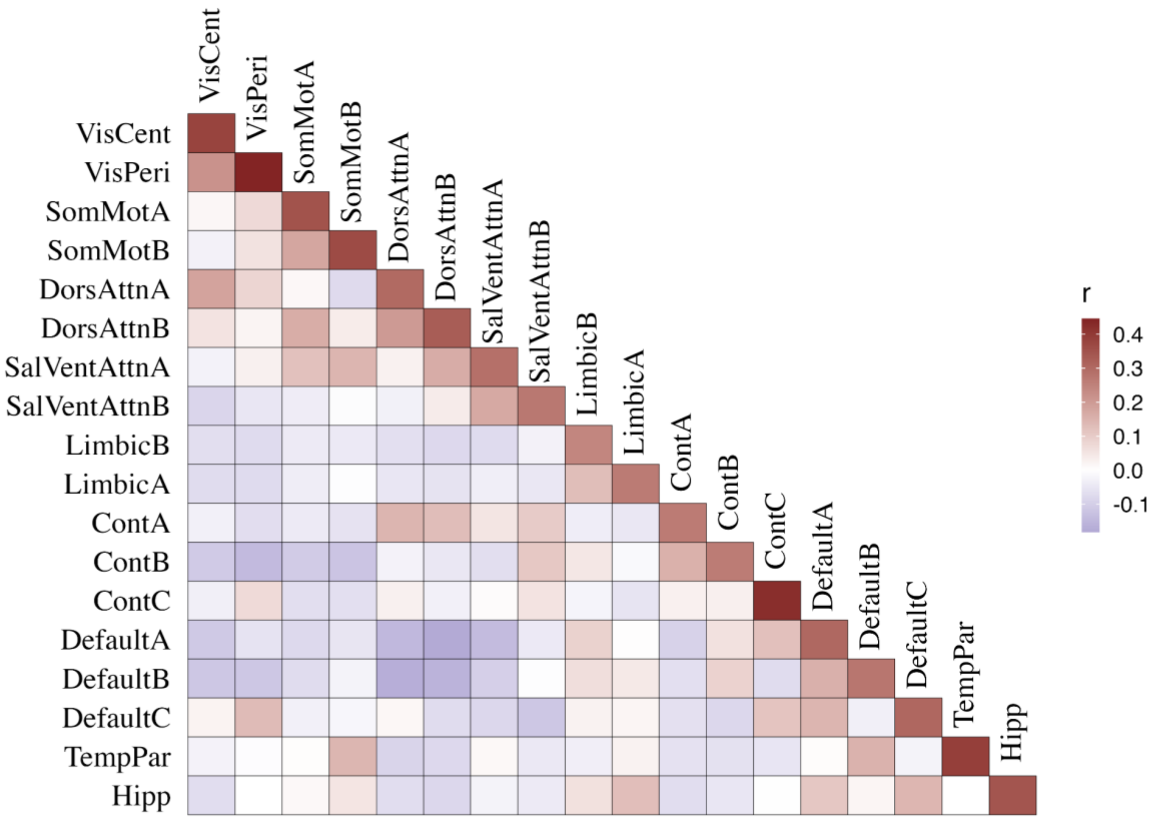

Figure S1: *Grand mean connectivity matrix.* Here we report the grand mean functional connectivity matrix, calculated by averaging functional connectivity estimates between our networks of interest across all subjects included in our analysis. As expected, regions included in the same functional network display increased functional connectivity. See Yeo et al. (2011) and Schaefer et al. (2018) for descriptions of each network.

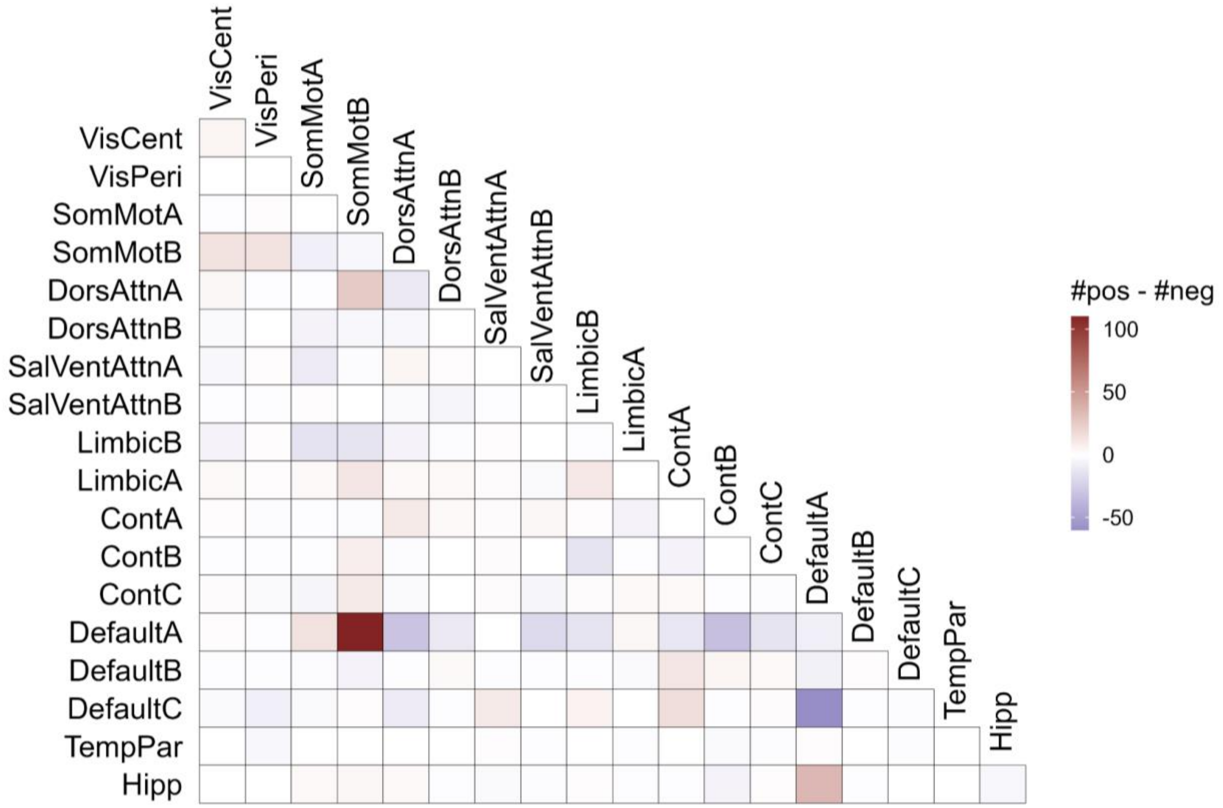

Figure S2: *Number of positive - negative connections that entered our predictive model*. For each pair of our networks of interest, we calculated the number of connections that positively correlated with memory ability at a $p < 0.01$ (our connection selection threshold, see text) and subtracted the number of connections that negatively correlated with memory ability at a $p < 0.01$.

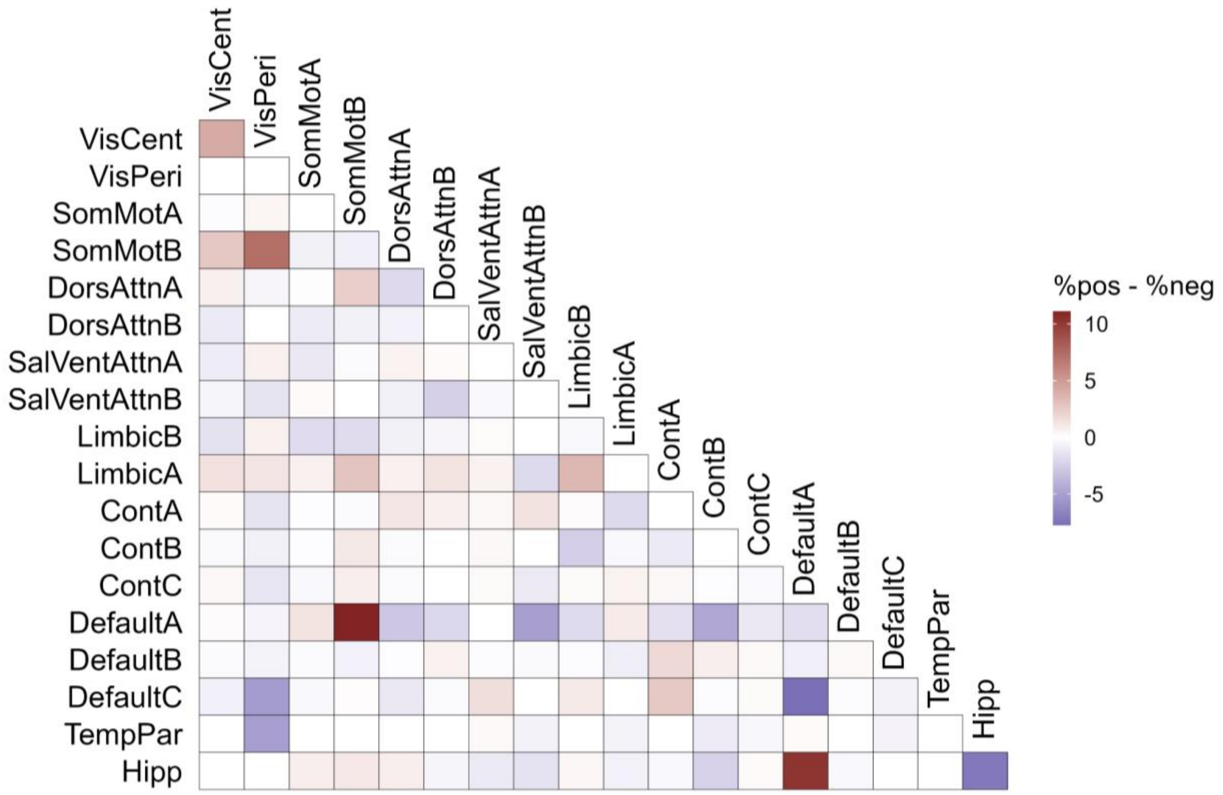

Figure S3: *Proportion of connections that entered our predictive model*. For each pair of our networks of interest, we calculated the proportion of connections (of all possible connections involving that pair) that positively correlated with memory ability at a $p < 0.01$ (our connection selection threshold, see text) and subtracted the proportion of connections that negatively correlated with memory ability at a $p < 0.01$.

Analyses excluding global signal regression

Global signal regression (GSR) involves statistically removing the global mean signal from the timecourse of each voxel in the brain prior to calculating functional connectivity between brain regions (see Murphy & Fox, 2017 for a review). GSR was originally thought to be an effective way to remove artifactual signals (e.g., motion) from voxel time series and was implemented in task-based fMRI experiments. An important caveat to this procedure is that it mathematically mandates negative connectivity estimates between brain regions (Murphy et al., 2009), muddying the interpretation of resulting anti-correlations. Recent research, however, suggests that GSR helps analyses whose aim is to predict behavior using functional connectivity estimates (Finn & Bandettini, 2021; Li et al., 2019), likely related to its efficacy in controlling head-related motion artifacts and other noise sources affecting the global signal (Satterthwaite et al., 2019). In our pre-registration, we did not include GSR in our preprocessing pipeline, though we have since been convinced by these recent studies that this step may be important for individual difference analyses. In the main text, we report results including GSR, and for completeness, we reran our analyses *excluding* GSR from our preprocessing pipeline and report the results below. Removing GSR had no impact on our pre-registered, hypothesis-driven analyses targeting the DMN-C (Supplemental Tables 1-3). However, there were two differences in the other results: First, removing GSR results in a statistically significant relationship between average hippocampal connectivity on memory ability, wherein average hippocampal connectivity was inversely related to memory ability (Supplemental Table 4). Second, removing GSR resulted in a substantially weaker ability to predict memory ability in the CBPM analysis ($r_{\{\text{observed, predicted}\}} = 0.081$, $p = 0.099$), controlling for age, sex, and framewise displacement. In other words, it appears that including GSR reduced the negative relationship between

hippocampal connectivity and memory, but it improved predictive performance in the overall CBPM analysis.

One possible explanation for this discrepancy is that, while GSR effectively minimizes the influence of head motion on functional connectivity estimates, it does so in a distance-dependent way (Satterthwaite et al., 2013), controlling for motion-related covariation among long-range connections more effectively than among short-range connections (Satterthwaite et al., 2019). We speculate without GSR, unaccounted motion artifacts, especially among long-range connections, might have resulted in a spurious connection between hippocampal connectivity and memory ability, while also interfering with the generalizability of predictions in the cross-validated CBPM analysis. Alternatively, there may be an actual trend toward a negative relationship between hippocampal connectivity and memory ability, as we also observed this relationship when using a composite score of memory ability (see Results) with GSR. This pattern would be unexpected, based on past research showing that hippocampal connectivity is positively related to memory ability (Setton et al., 2022; Touroutoglou et al., 2015; Wang, LaViolette, et al., 2010; Wang, Negreira, et al., 2010). However, in one study using a relatively small sample, there was evidence for a negative relationship between memory detail and hippocampal functional connectivity, specifically with the medial prefrontal cortex, but only among older adults (Matijevic et al., 2022). While it is important to consider the full spectrum of possible results here, due to the unexpectedness of this result and its sensitivity to different analysis decisions, we think that it is best to remain cautious in interpreting this result.

	Model 1		Model 2		Model 3	
**Characteristic**	**Beta**[1]	**SE**[2]	**Beta**[1]	**SE**[2]	**Beta**[1]	**SE**[2]
within	-0.29	0.225	-0.27	0.225	-0.18	0.204
age			-0.05	0.027	-0.05	0.026
sex[3]			-0.91*	0.446	-1.00*	0.414
fd			-6.36	5.70	1.98	5.21
acer					1.10***	0.243
cattell					0.83***	0.237
No. Obs.	243		243		235	
R^2	0.007		0.048		0.238	

Table S1: Regression results of average within DMN-C connectivity on memory ability removing GSR from our analysis pipeline. within = average strength of connection among DMN-C regions; acer = cognitive function score, cattell = fluid intelligence score. [1]*p<0.05; **p<0.01; ***p<0.001. [2]SE = Standard Error. [3]Female = 0, Male = 1.

	Model 4		Model 5		Model 6	
**Characteristic**	**Beta**[1]	**SE**[2]	**Beta**[1]	**SE**[2]	**Beta**[1]	**SE**[2]
between	-0.35	0.224	-0.22	0.231	-0.07	0.208
age			-0.04	0.027	-0.05	0.026
sex[3]			-0.95*	0.444	-1.02*	0.414
fd			-5.62	5.86	1.93	5.33
acer					1.11***	0.244
cattell					0.82***	0.237
No. Obs.	243		243		235	
R^2	0.010		0.046		0.235	

Table S2: Regression results of average DMN-C--DMN-A connectivity on memory ability removing GSR from our analysis pipeline. between = average strength of connection between DMN-C and DMN-A regions; acer = cognitive function score, cattell = fluid intelligence score. [1]*p<0.05; **p<0.01; ***p<0.001. [2]SE = Standard Error. [3]Female = 0, Male = 1.

	Model 7		Model 8		Model 9	
**Characteristic**	**Beta**[1]	**SE**[2]	**Beta**[1]	**SE**[2]	**Beta**[1]	**SE**[2]
extra	-0.60**	0.222	-0.40	0.252	-0.31	0.230
age			-0.04	0.028	-0.04	0.026
sex[3]			-0.85	0.449	-0.94*	0.417
fd			-3.21	6.15	4.35	5.57
acer					1.09***	0.243
cattell					0.84***	0.237
No. Obs.	243		243		235	
R^2	0.030		0.052		0.241	

Table S3: Regression results of average DMN-C connectivity with the rest of the brain on memory ability removing GSR from our analysis pipeline. extra = average strength of connection between DMN-C regions and regions not in the DMN-C or DMN-A; acer = cognitive function score, cattell = fluid intelligence score. [1]*p<0.05; **p<0.01; ***p<0.001. [2]SE = Standard Error. [3]Female = 0, Male = 1.

	Model 10		Model 11		Model 12	
**Characteristic**	**Beta**[1]	**SE**[2]	**Beta**[1]	**SE**[2]	**Beta**[1]	**SE**[2]
hipp	-0.74***	0.220	-0.57*	0.241	-0.44*	0.218
age			-0.04	0.027	-0.04	0.026
sex[3]			-0.73	0.451	-0.84*	0.421
fd			-2.39	5.95	4.77	5.39
acer					1.09***	0.242
cattell					0.81***	0.235
No. Obs.	243		243		235	
R^2	0.044		0.065		0.249	

Table S4: Regression results of average hippocampal connectivity on memory ability removing GSR from our analysis pipeline. hipp = average strength of connection of hippocampal regions; acer = cognitive function score, cattell = fluid intelligence score. [1]*p<0.05; **p<0.01; ***p<0.001. [2]SE = Standard Error. [3]Female = 0, Male = 1.

Impact of the connection selection threshold used in CBPM

Our study used Connectome Based Predictive Modeling (CBPM; Shen et al., 2017) to determine if information contained within the functional connectome is useful for predicting memory ability. This approach involves setting an arbitrary threshold for defining which connections are used to make behavioral predictions. Analyses reported in this manuscript used a connection selection threshold of $p <= .01$. It is unclear, however, whether this arbitrary choice of threshold has any impact on our results. We reran our key CBPM analysis (i.e., predicting memory ability while controlling for age, sex, and average framewise displacement) using a range of connection selection thresholds: p = [0.001 0.005 0.01 0.05 0.1]. Results are reported in Supplemental Figure 4. Analyses using all selection thresholds were statistically significant with one exception – when the selection threshold was set to $p < 0.005$. It is currently unclear why this specific analysis failed — selecting an even stricter threshold (i.e., $p < 0.001$) resulted in restored predictive performance. We take this pattern of results as evidence that the CBPM approach is robust to connection selection threshold, in line with previous reports (Finn et al., 2015; Jangraw et al., 2018; Shen et al., 2017).

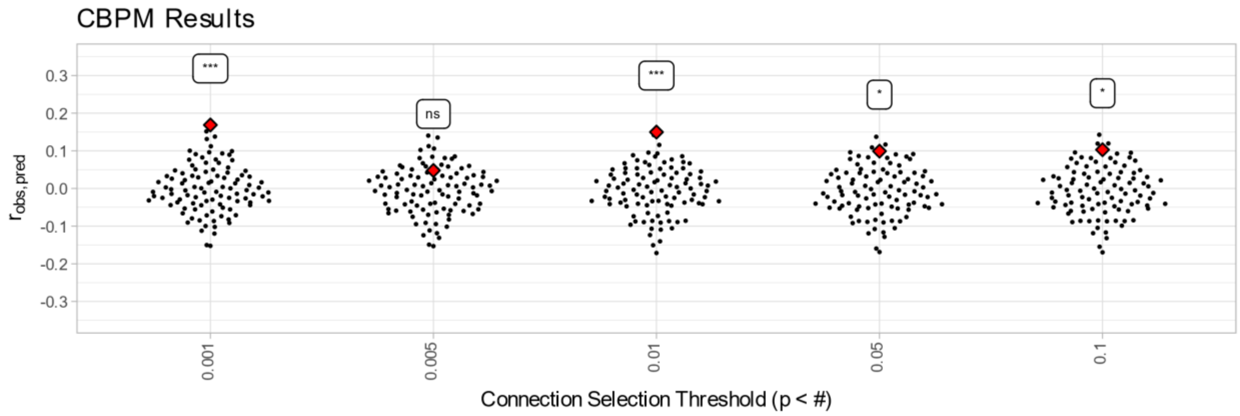

Figure S4: CBPM results are robust to selection of connection selection threshold in the CBPM analysis. Red diamonds indicate observed results, black dots indicate results of 100 null simulations. *** $p <= 0.001$, ** $p <= 0.01$, * $p <= 0.05$, ns = $p > 0.05$.

Combining data across tasks appears to improve our ability to predict memory ability out of sample.

Our analyses combined data across tasks to increase the reliability of our functional connectivity estimates (Elliott et al., 2019). This approach assumes that intrinsic functional connectivity would not vary substantially across tasks. Indeed, a recent study suggests that variability in the functional connectome is dominated by a normative pattern, patterns unique to individuals, and patterns unique to how individuals complete certain tasks (Gratton et al., 2018). To test the validity of this approach, we calculated the functional connectome separately for each task for each subject (i.e., movie watching, rest, sensorimotor) and correlated the resulting task-specific functional connectomes. Supplemental Table 5 displays the mean, standard deviation, minimum, and maximum similarity for each pair of tasks across our sample of 243 subjects. All correlations between tasks were performed on the subset of subjects that had a pair of valid scans. The movie-watching connectome was notably less similar to the rest and sensorimotor

task connectomes. We suspect that this could be due to stimulus-driven changes in brain activation.

Task Pair	N	min	max	mean	sd
movie-smt	240	0.20	0.69	0.51	0.06
rest-movie	227	0.18	0.66	0.49	0.06
rest-smt	226	0.47	0.79	0.63	0.07

Table S5: How similar are connectomes calculated using data from different tasks? Similarity between connectomes was calculated using a Pearson's correlation. N = number of subjects with valid scans for both scans in this pair, min = minimum Peasrson's correlation between connectomes in task pair, max = maximum Pearson's correlation between connectomes in task pair, mean = average Pearson's correlation between connectomes in task pair.

To see how this impacted our results, we reran our CBPM analyses using functional connectomes calculated using only the movie-watching data ("movie"), only the resting-state data ("rest"), and only the sensorimotor ("smt") tasks data. The results of these CBPM analyses are reported in Supplemental Figure 5. Functional connectomes calculated using the resting-state ($r_{\{observed, predicted\}}$ = -0.007, p = 0.39) and sensorimotor task ($r_{\{observed, predicted\}}$ = 0.083, p = 0.12) data were insufficient for predicting memory ability. Functional connectomes calculated using the movie watching data, however, were sufficient for predicting memory ability ($r_{\{observed, predicted\}}$ = 0.113, p = 0.03). Interestingly, all the task specific predictive models performed worse compared with our predictive model that used a combined "intrinsic" connectome for each subject by averaging across tasks ($r_{\{observed, predicted\}}$ = 0.1498, p < 0.01).

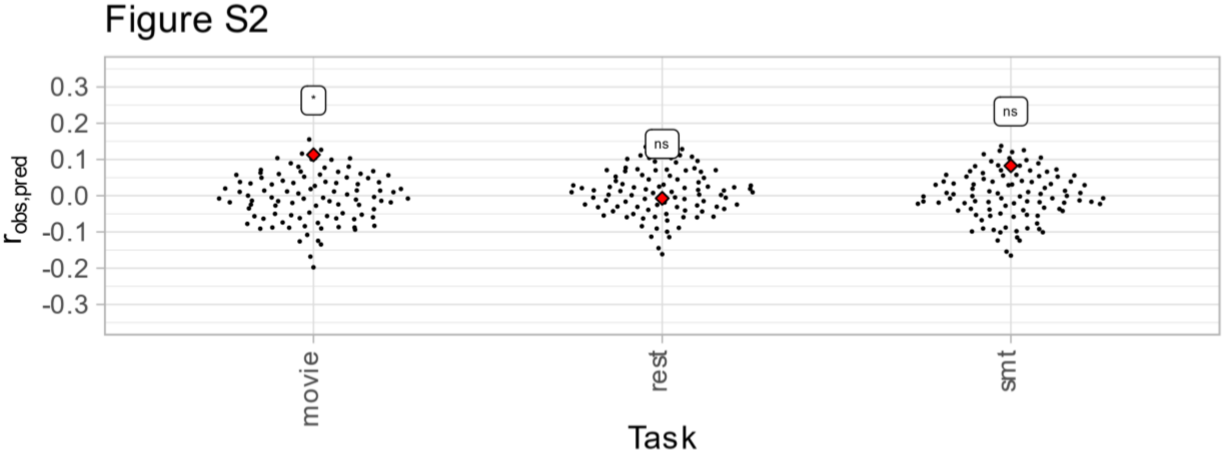

Figure S5: How do predictive models built using connectomes from individual tasks compare to models built using an "intrinsic" connectome, calculated by averaging across tasks? Movie = movie watching, rest = resting-state, smt = sensorimotor task.

Detailed description of the fMRIPrep pipeline

The following is an edited version of the recommended boilerplate output by *fMRIPrep* after processing our data. The original boilerplate contained redundant descriptions of the operations performed by the software. What appears below is a detailed description of the processing steps with the redundant descriptions removed. Results included in this manuscript come from preprocessing performed using *fMRIPrep 20.2.0* (Esteban, Markiewicz, et al. (2018); Esteban, Blair, et al. (2018); RRID:SCR_016216), which is based on *Nipype 1.5.1* (Gorgolewski et al. (2011); Gorgolewski et al. (2018); RRID:SCR_002502).

Anatomical data preprocessing

A total of 1 T1-weighted (T1w) images were found within the input BIDS dataset. The T1-weighted (T1w) image was corrected for intensity non-uniformity (INU) with **N4BiasFieldCorrection** (Tustison et al. 2010), distributed with ANTs 2.3.3 (Avants et al. 2008, RRID:SCR_004757), and used as T1w-reference throughout the workflow. The T1w-reference

was then skull-stripped with a *Nipype* implementation of the **antsBrainExtraction.sh** workflow (from ANTs), using OASIS30ANTs as target template. Brain tissue segmentation of cerebrospinal fluid (CSF), white-matter (WM) and gray-matter (GM) was performed on the brain-extracted T1w using **fast** (FSL 5.0.9, RRID:SCR_002823, Zhang, Brady, and Smith 2001). Volume-based spatial normalization to one standard space (MNI152NLin2009cAsym) was performed through nonlinear registration with **antsRegistration** (ANTs 2.3.3), using brain-extracted versions of both T1w reference and the T1w template. The following template was selected for spatial normalization: *ICBM 152 Nonlinear Asymmetrical template version 2009c* [Fonov et al. (2009), RRID:SCR_008796; TemplateFlow ID: MNI152NLin2009cAsym].

Functional data preprocessing

For each of the 3 BOLD runs found per subject (across all tasks and sessions), the following preprocessing was performed. First, a reference volume and its skull-stripped version were generated from the shortest echo of the BOLD run using a custom methodology of *fMRIPrep*. A B0-nonuniformity map (or *fieldmap*) was estimated based on a phase-difference map calculated with a dual-echo GRE (gradient-recall echo) sequence, processed with a custom workflow of *SDCFlows* inspired by the epidewarp.fsl script and further improvements in HCP Pipelines (Glasser et al. 2013). The *fieldmap* was then co-registered to the target EPI (echo-planar imaging) reference run and converted to a displacements field map (amenable to registration tools such as ANTs) with FSL's **fugue** and other *SDCflows* tools. Based on the estimated susceptibility distortion, a corrected EPI (echo-planar imaging) reference was calculated for a more accurate co-registration with the anatomical reference. The BOLD reference was then co-registered to the T1w reference using **flirt** (FSL 5.0.9, Jenkinson and Smith 2001) with the boundary-based registration (Greve and Fischl 2009) cost-function. Co-

registration was configured with nine degrees of freedom to account for distortions remaining in the BOLD reference. Head-motion parameters with respect to the BOLD reference (transformation matrices, and six corresponding rotation and translation parameters) are estimated before any spatiotemporal filtering using **mcflirt** (FSL 5.0.9, Jenkinson et al. 2002). BOLD runs were slice-time corrected using **3dTshift** from AFNI 20160207 (Cox and Hyde 1997, RRID:SCR_005927). The BOLD time-series (including slice-timing correction when applied) were resampled onto their original, native space by applying a single, composite transform to correct for head-motion and susceptibility distortions. These resampled BOLD time-series will be referred to as *preprocessed BOLD in original space*, or just *preprocessed BOLD*. A T2* map was estimated from the preprocessed BOLD by fitting to a monoexponential signal decay model with nonlinear regression, using T2*/S0 estimates from a log-linear regression fit as initial values. For each voxel, the maximal number of echoes with reliable signal in that voxel were used to fit the model. The calculated T2* map was then used to optimally combine preprocessed BOLD across echoes following the method described in (Posse et al. 1999). The optimally combined time series was carried forward as the *preprocessed BOLD*. The BOLD time-series were resampled into standard space, generating a *preprocessed BOLD run in MNI152NLin2009cAsym space*. Several confounding time-series were calculated based on the *preprocessed BOLD*: framewise displacement (FD), DVARS and three region-wise global signals. FD was computed using two formulations following Power (absolute sum of relative motions, Power et al. (2014)) and Jenkinson (relative root mean square displacement between affines, Jenkinson et al. (2002)). FD and DVARS are calculated for each functional run, both using their implementations in *Nipype* (following the definitions by Power et al. 2014). The three global signals are extracted within the CSF, the WM, and the whole-brain masks. Additionally, a

set of physiological regressors were extracted to allow for component-based noise correction (*CompCor*, Behzadi et al. 2007). Principal components are estimated after high-pass filtering the preprocessed BOLD time-series (using a discrete cosine filter with 128s cut-off) for the two *CompCor* variants: temporal (tCompCor) and anatomical (aCompCor). tCompCor components are then calculated from the top 2% variable voxels within the brain mask. For aCompCor, three probabilistic masks (CSF, WM and combined CSF+WM) are generated in anatomical space. The implementation differs from that of Behzadi et al. in that instead of eroding the masks by 2 pixels on BOLD space, the aCompCor masks are subtracted a mask of pixels that likely contain a volume fraction of GM. This mask is obtained by thresholding the corresponding partial volume map at 0.05, and it ensures components are not extracted from voxels containing a minimal fraction of GM. Finally, these masks are resampled into BOLD space and binarized by thresholding at 0.99 (as in the original implementation). Components are also calculated separately within the WM and CSF masks. For each CompCor decomposition, the *k* components with the largest singular values are retained, such that the retained components' time series are sufficient to explain 50 percent of variance across the nuisance mask (CSF, WM, combined, or temporal). The remaining components are dropped from consideration. The head-motion estimates calculated in the correction step were also placed within the corresponding confounds file. The confound time series derived from head motion estimates and global signals were expanded with the inclusion of temporal derivatives and quadratic terms for each (Satterthwaite et al. 2013). Frames that exceeded a threshold of 0.5 mm FD or 1.5 standardized DVARS were annotated as motion outliers. All resamplings can be performed with *a single interpolation step* by composing all the pertinent transformations (i.e. head-motion transform matrices, susceptibility distortion correction when available, and co-registrations to anatomical and output

spaces). Gridded (volumetric) resamplings were performed using **antsApplyTransforms** (ANTs), configured with Lanczos interpolation to minimize the smoothing effects of other kernels (Lanczos 1964). Non-gridded (surface) resamplings were performed using **mri_vol2surf** (FreeSurfer).

Many internal operations of *fMRIPrep* use *Nilearn* 0.6.2 (Abraham et al. 2014, RRID:SCR_001362), mostly within the functional processing workflow. For more details of the pipeline, see the fMRIPrep documentation (https://fmriprep.org/en/20.2.0/workflows.html).

Supplemental References

Elliott, M. L., Knodt, A. R., Cooke, M., Kim, M. J., Melzer, T. R., Keenan, R., Ireland, D., Ramrakha, S., Poulton, R., Caspi, A., Moffitt, T. E., & Hariri, A. R. (2019). General functional connectivity: Shared features of resting-state and task fMRI drive reliable and heritable individual differences in functional brain networks. *NeuroImage*, *189*, 516–532. https://doi.org/10.1016/j.neuroimage.2019.01.068

Finn, E. S., & Bandettini, P. A. (2021). Movie-watching outperforms rest for functional connectivity-based prediction of behavior. *NeuroImage*, *235*, 117963. https://doi.org/10.1016/j.neuroimage.2021.117963

Finn, E. S., Shen, X., Scheinost, D., Rosenberg, M. D., Huang, J., Chun, M. M., Papademetris, X., & Constable, R. T. (2015). Functional connectome fingerprinting: identifying individuals using patterns of brain connectivity. *Nature Neuroscience, 18*(11), 1664–1671. https://doi.org/10.1038/nn.4135

Gratton, C., Laumann, T. O., Nielsen, A. N., Greene, D. J., Gordon, E. M., Gilmore, A. W., Nelson, S. M., Coalson, R. S., Snyder, A. Z., Schlaggar, B. L., Dosenbach, N. U. F., & Petersen, S. E. (2018). Functional Brain Networks Are Dominated by Stable Group and Individual Factors, Not Cognitive or Daily Variation. *Neuron*, *98*(2), 439–452.e5. https://doi.org/10.1016/j.neuron.2018.03.035

Jangraw, D. C., Gonzalez-Castillo, J., Handwerker, D. A., Ghane, M., Rosenberg, M. D., Panwar, P., & Bandettini, P. A. (2018). A functional connectivity-based neuromarker of sustained attention generalizes to predict recall in a reading task. *NeuroImage*, *166*, 99–109. https://doi.org/10.1016/j.neuroimage.2017.10.019

Li, J., Kong, R., Liégeois, R., Orban, C., Tan, Y., Sun, N., Holmes, A. J., Sabuncu, M. R., Ge, T., & Yeo, B. T. T. (2019). Global signal regression strengthens association between resting-state functional connectivity and behavior. *NeuroImage*, *196*, 126–141. https://doi.org/10.1016/j.neuroimage.2019.04.016

Matijevic, S., Andrews-Hanna, J. R., Wank, A. A., Ryan, L., & Grilli, M. D. (2022). Individual differences in the relationship between episodic detail generation and resting state functional connectivity vary with age. *Neuropsychologia*, *166*, 108138. https://doi.org/10.1016/j.neuropsychologia.2021.108138

Murphy, K., Birn, R. M., Handwerker, D. A., Jones, T. B., & Bandettini, P. A. (2009). The impact of global signal regression on resting state correlations: are anti-correlated networks introduced? *NeuroImage*, *44*(3), 893–905. https://doi.org/10.1016/j.neuroimage.2008.09.036

Murphy, K., & Fox, M. D. (2017). Towards a consensus regarding global signal regression for resting state functional connectivity MRI. *NeuroImage*, *154*, 169–173. https://doi.org/10.1016/j.neuroimage.2016.11.052

Satterthwaite, T. D., Ciric, R., Roalf, D. R., Davatzikos, C., Bassett, D. S., & Wolf, D. H. (2019). Motion artifact in studies of functional connectivity: Characteristics and mitigation strategies. *Human Brain Mapping*, *40*(7), 2033–2051. https://doi.org/10.1002/hbm.23665

Satterthwaite, T. D., Elliott, M. A., Gerraty, R. T., Ruparel, K., Loughead, J., Calkins, M. E., Eickhoff, S. B., Hakonarson, H., Gur, R. C., Gur, R. E., & Wolf, D. H. (2013). An improved framework for confound regression and filtering for control of motion artifact in the preprocessing of resting-state functional connectivity data. *NeuroImage*, *64*, 240–256. https://doi.org/10.1016/j.neuroimage.2012.08.052

Schaefer, A., Kong, R., Gordon, E. M., Laumann, T. O., Zuo, X.-N., Holmes, A. J., Eickhoff, S. B., & Yeo, B. T. T. (2018). Local-Global Parcellation of the Human Cerebral Cortex from Intrinsic Functional Connectivity MRI. *Cerebral Cortex*, *28*(9), 3095–3114. https://doi.org/10.1093/cercor/bhx179

Setton, R., Mwilambwe-Tshilobo, L., Sheldon, S., Turner, G. R., & Spreng, R. N. (2022). Hippocampus and temporal pole functional connectivity is associated with age and individual differences in autobiographical memory. *Proceedings of the National Academy of Sciences of the United States of America*, *119*(41), e2203039119. https://doi.org/10.1073/pnas.2203039119

Shen, X., Finn, E. S., Scheinost, D., Rosenberg, M. D., Chun, M. M., Papademetris, X., & Constable, R. T. (2017). Using connectome-based predictive modeling to predict individual behavior from brain connectivity. *Nature Protocols*, *12*(3), 506–518. https://doi.org/10.1038/nprot.2016.178

Touroutoglou, A., Andreano, J. M., Barrett, L. F., & Dickerson, B. C. (2015). Brain network connectivity-behavioral relationships exhibit trait-like properties: Evidence from hippocampal connectivity and memory. *Hippocampus*, *25*(12), 1591–1598. https://doi.org/10.1002/hipo.22480

Wang, L., LaViolette, P., O'Keefe, K., Putcha, D., & Bakkour, A. (2010). Intrinsic connectivity between the hippocampus and posteromedial cortex predicts memory performance in cognitively intact older individuals. *NeuroImage*. https://www.sciencedirect.com/science/article/pii/S1053811910002144

Wang, L., Negreira, A., LaViolette, P., Bakkour, A., Sperling, R. A., & Dickerson, B. C. (2010). Intrinsic interhemispheric hippocampal functional connectivity predicts individual differences in memory performance ability. *Hippocampus*, *20*(3), 345–351. https://doi.org/10.1002/hipo.20771
